# Impact of water stress under ambient and elevated carbon dioxide across three temperature regimes on soybean canopy gas exchange and productivity

**DOI:** 10.1038/s41598-021-96037-9

**Published:** 2021-08-13

**Authors:** Shardendu K. Singh, Vangimalla R. Reddy, Mura Jyostna Devi, Dennis J. Timlin

**Affiliations:** 1 Adaptive Cropping Systems Laboratory, USDA-ARS, Beltsville, MD USA; 2 Vegetable Crops Research Unit, USDA-ARS, Department of Horticulture, University of Wisconsin-Madison, Madison, WI USA; 3 Present Address: AeroFarms, Newark, NJ USA

**Keywords:** Photosynthesis, Plant development, Plant physiology, Plant stress responses, Plant sciences

## Abstract

The present study investigated the interactive effects of three environmental stress factors elevated CO_2_, temperature, and drought stress on soybean growth and yield. Experiments were conducted in the sunlit, controlled environment Soil–Plant–Atmosphere–Research chambers under two-level of irrigation (WW-well water and WS-water stress-35%WW) and CO_2_ (aCO_2-_ambient 400 µmol mol^−1^ and eCO_2_-elevated 800 µmol mol^−1^) and each at the three day/night temperature regimes of 24/18 °C (MLT-moderately low), 28/22 °C (OT-optimum), and 32/26 °C (MHT-moderately high). Results showed the greatest negative impact of WS on plant traits such as canopy photosynthesis (*P*_Cnet_), total dry weight (TDwt), and seed yield. The decreases in these traits under WS ranged between 40 and 70% averaged across temperature regimes with a greater detrimental impact in plants grown under aCO_2_ than eCO_2_. The MHT had an increased *P*_Cnet_, TDwt, and seed yield primarily under eCO_2,_ with a greater increase under WW than WS conditions. The eCO_2_ stimulated *P*_Cnet_, TDwt, and seed yield more under WS than WW. For instance, on average across T regimes, eCO_2_ stimulated around 25% and 90% dry mass under WW and WS, respectively, relative to aCO_2_. Overall, eCO_2_ appears to benefit soybean productivity, at least partially, under WS and the moderately warmer temperature of this study.

## Introduction

The impact of seasonal weather patterns is likely to be greater on crop productivity when it is combined with climate change. The average global air temperature has increased in the past decades by 0.4 °C and likely to exceed 1.5 °C by the end of the twenty-first century^1^. Temperatures below or above the optimum often result in loss of crop yield due to rate-limited photosynthesis or reduced vegetative and reproductive growth^2–4^. Increases in the frequency and intensity of extreme events such as heatwaves and drought have also been predicted^4,5^. Without CO_2_ fertilization and effective adaptations, each degree-Celsius increase in global mean temperature would, on average, reduce global soybean yields by 3.1%^6^. In the natural condition, crops are simultaneously exposed to the interaction among multiple abiotic and biotic factors. The interactive impact of stress factors on crop plants is complex, and a better understanding of crop systems is required to predict their response to the changing environment^7,8^.

The prevailing notion is that the elevated CO_2_ will enhance soybean productivity^9^. However, elevated CO_2_-mediated increases in soybean yield are highly suppressed under temperature and water stress due to the adverse effects on photosynthesis, flowering, pod set, pollen viability, and seed development^10–12^. Moreover, the combined effects of two or more stress factors (e.g., drought and temperature) have been reported to be more deleterious than the effects of a single stress factor on crop productivity, including soybean^7,13,14^. Since elevated CO_2_ stimulates photosynthesis, the beneficial effects of elevated CO_2_ can still be expected at least under moderate stress conditions due to an increased energy (carbohydrate) supply and the potential conservation of soil water^7,15,16^. However, a thorough investigation of this idea is constrained by the need for a state-of-the-art facility in multiple numbers to precisely control and allocate the combinations of the environmental factors (e.g., levels of temperature, water, and CO_2_).

The CO_2_, temperature, and water availability strongly affect carbon gain resulting from photosynthesis. Photosynthesis contributes to the majority of plant dry matter, consisting of ≈ 96% carbon, hydrogen, and oxygen. Plant tissue contains approximately 40–50% of carbon, and it is primarily derived from photosynthesis^17–19^. The experimental data on the quantification of grain crops such as soybean to the interaction of CO_2_, temperature, and water availability is limited^11,14,20,21^. Although interaction studies have advanced our understanding of crop response to multiple environments, the use of a chronic high temperature or severe water stress is not rare. Moreover, investigations on the interaction of drought together with the CO_2_ and temperature on soybean are extremely limited^22,23^. Depending on the location and year, soybean is often grown under both the limited-environment where stress situations are persistent moderately throughout the growing season or in the non-limited environment where episodes of heat and drought stresses occur during the critical growth periods. Therefore, it is essential to quantify soybean response under the long-term moderate abiotic stresses. The progress in the investigation of crop response to the combination of environmental constraints is slow due to the need for state-of-the-art facilities where the choice of treatment combinations can be used to address a set of specific questions.

The current study was designed to investigate the interactive impacts of water availability with CO_2_ and temperature in the state-of-the-art facility that is sunlit and controlled environment Soil-Plant-Atmosphere-Research (SPAR) chambers. The objective was to quantify the interactive effects of irrigation amount, air CO_2_, and air temperature on soybean gas exchange, biomass accumulation, and seed yield. The hypotheses were (i) impacts of water stress will be more detrimental under ambient than elevated CO_2_ and (ii) a moderately warmer temperature will enhance soybean productivity, especially under well-watered conditions.

## Results and discussion

### The irrigation amount almost matched the desired WS

A 100% seed emergence was observed seven days after planting. Soybean plants in all treatments were at R1 stage (flowering) between 40 and 44 days after planting. The soil in each treatment was saturated during the filling and held approximately on average ≈ 180 ± 11 and 160 ± 8 L of water that was readily available to the plants in WW and WS treatments, respectively. Throughout the growing season, sufficient water was available across WW treatments as deduced from the hourly dynamics profile of soil water (Supplementary Fig. S1). The water availability gradually depleted in WS treatments and ≈ 58 DAE (8/15/2018) appeared to stabilize, indicating a balance between irrigation and plant water use. During the initial growth period under WS treatments, relatively faster depletion of the soil water was also apparent as T increased across CO_2_ levels. However, such water depletion from soil was relatively slower for eCO_2_ versus aCO_2_. This is most likely caused by a well-known plant response to eCO_2_ that the decreased stomatal conductance reduces water use and conserves soil moisture^11^. At the end of the growing season, the total irrigation for WS treatments expressed as a percentage of the WW control for the corresponding CO_2_ treatment at each T regime was between 32 and 36%, close to the desired 35% (Supplementary Table S1).

### Plant water use: sessional water use efficiency increased mainly under eCO_2_ across irrigation regimes

**Figure 1:**
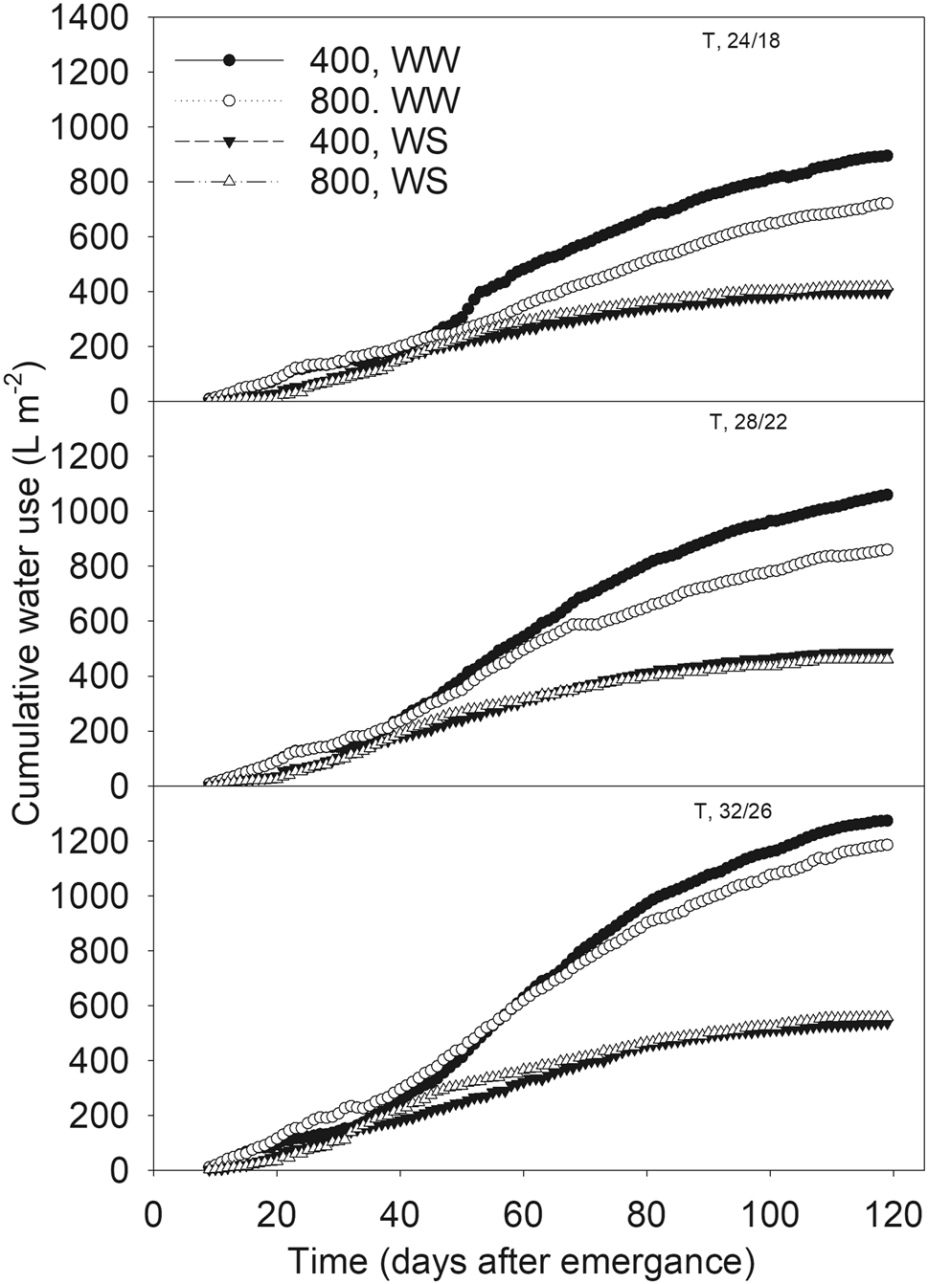
The daily water use between 6/26/2018 (8 days after emergence) and the maturity of soybean across treatments of two levels of CO_2_ (400 and 800 µmol mol^−1^) and irrigation (WW, well water; WS, water stress) across three temperature (T, day/night, °C) regimes.

The total uptake exceeded the total irrigation as shown in Supplementary Table S1 because of the utilization of the available soil water added at the planting. WW treatments showed 19% (under MLT and OT) and 7% (under MHT) lesser seasonal water use for eCO_2_ versus aCO_2_ (Supplementary Table S1). This was also apparent from the clear pattern of the cumulative water use between CO_2_ levels throughout the season (Fig. 1). As also shown in this study, the extent of transpiration driven by stomatal conductance decreases under eCO_2_ conserving water while stimulation of photosynthesis and biomass accumulation results into greater water use efficiency^24^. A distinctive seasonal pattern between CO_2_ levels could not be observed within WS treatments under given temperature treatments, indicating the dominating impact of water availability (Fig. 1). Regardless of the irrigation or CO_2_ treatments, the seasonal water use was either lesser at cooler (MLT) or greater at warmer (MHT) temperature relative to the OT (Supplementary Table S1).

**Figure 2:**
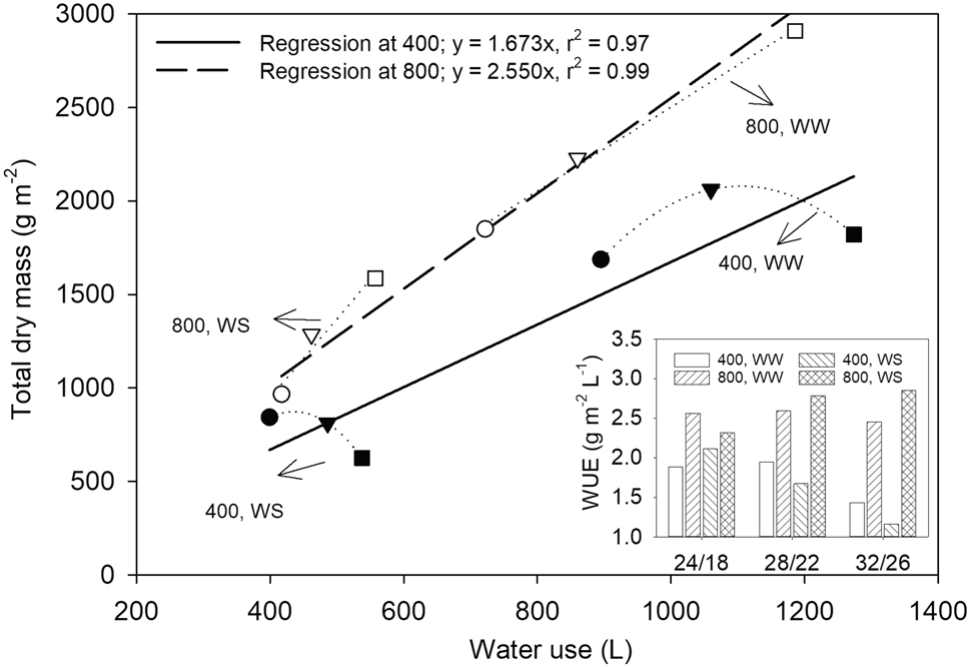
The total dry mass production (sum of the three harvests) as a function of the total water use over the growing period. The solid and dashed lines show the linear regression across temperature and irrigation treatments for two CO_2_ levels separately. The dotted lines show either the polynomial 2nd order regression fit at ambient CO_2_ or linear fit at elevated CO_2_ for each irrigation treatment across the three day/night temperature regimes (circle, 24/18; triangle, 28/22; square 32/26 °C). Filled and unfilled symbols represent the ambient and elevated CO_2_, respectively. The bar graph at the lower right corner shows the water use efficiency (WUE, g dry mass m^−2^ L^−1^ of water) as influenced by CO_2_ levels (400 and 800 µmol mol^−1^) and irrigation (WW, well water; WS, water stress) under three temperature (T, day/night, °C) regimes. WUE was calculated as the ratio between total dry mass and water use.

**Figure 3:**
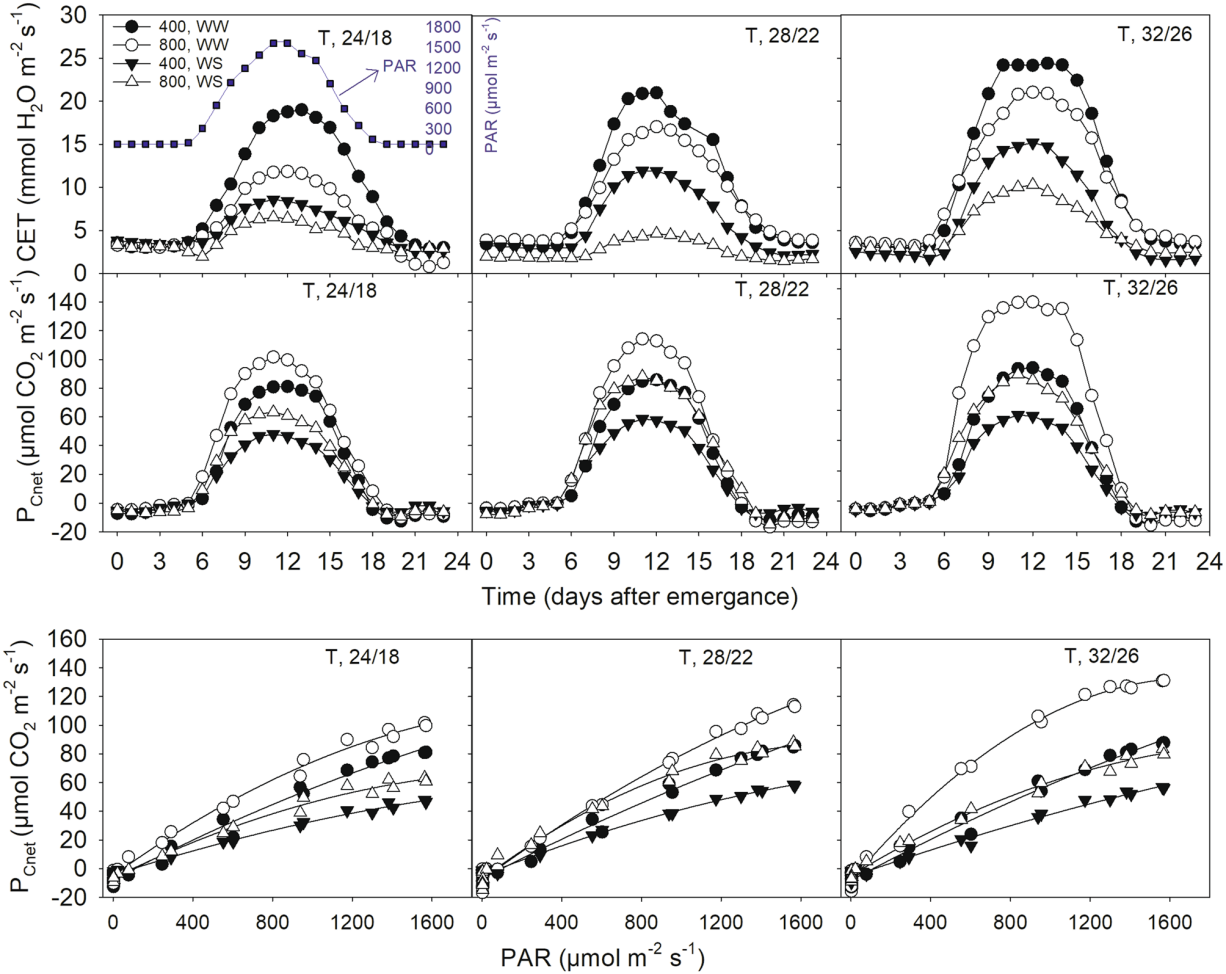
Daily soybean canopy net photosynthesis rates (*P*_Cnet_), canopy evapotranspiration (CET) and *P*_Cnet_ light response curve for a representative sunny day 58 days after emergence) as influenced by two levels of CO_2_ (400 and 800 µmol mol^−1^) and irrigation (WW, well water; WS, water stress) across three levels of temperature (T, day/night, °C). Datapoints represent the measured hourly mean. The diel photosynthetically active radiation (PAR, square symbols) for the day is also shown for the same day (top left panel). The *P*_Cnet_ response to light (PAR) is shown in the bottom panel.

To assess the water use efficiency (WUE, g dry mass m^−2^ L^−1^ water), the seasonal total dry mass production was regressed with the total water use by CO_2_ levels and by irrigation treatments (Fig. 2). The slope of the linear regression fit resulted in WUE of 1.673 and 2.55 g dry mass m^−2^ for each L of water at aCO_2_ and eCO_2_, respectively. Subsequently, the relationship between dry mass and water use appeared to strongly depend on T and irrigation treatments showing a curvilinear (polynomial second order) response at aCO_2_ and resulted in a downward trend as T increased. Warmer temperature drives greater transpirational use of water (e.g., CET) and can increase photosynthesis and biomass accumulation under moderately warm temperature resulting in greater water use efficiency^11,24^ (Fig. 3). The highest ratio between dry mass and water use was under eCO_2_ for WS at MHT (Fig. 2). An increased WUE in soybean under eCO_2_ is attributed to the stomatal closure induced by eCO_2_ while stimulating photosynthesis. Increased WUE under eCO_2_ and with or without a moderately warmer temperature has also been reported in soybean grown under open-top chambers^25^.

### Canopy gas exchange: CET decreased under WS and eCO_2_ while ***P***_Cnet_ positively responded to eCO_2_ across irrigation and T regimes

Over 90% of the plant biomass is derived from photosynthesis. Therefore, a close relationship is likely between CER and plant biomass production^26^. The canopy *P*_Cnet_ measured in this study from the SPAR chambers provides a non-destructive method for determining carbon fixed throughout the season^27,28^. The diel gas exchange (*P*_Cnet_ and CET) peaked during the midday and almost mimicked the sunlight intensity (PAR) from morning through evening hours (Fig. 3). The midday CET was always smaller at eCO_2_ versus aCO_2_ but greater at the warmer temperature. This can directly be attributed to the reduced transpiration due to stomatal closure under eCO_2_ while increased transpirational demand under warmer temperature^11,24^. In contrast, *P*_Cnet_ was greater at eCO_2_ regardless of the T or irrigation treatments (Fig. 3), and was in agreement with previously observed greater leaf or canopy photosynthesis under moderately warmer than optimum temperature with or without CO_2_ enrichment^25,29,30^. The eCO_2_ stimulates *P*_Cnet_ due to increase due to increased internal CO_2_ concentration inside the leaves^24^. The light response curves of *P*_Cnet_ derived from the diurnal CER measurements exhibited the treatment differences even at lower PAR levels (e.g., 500 µmol m^−2^ s^−1^) indicating photosynthetic sensitivity of soybean to the WS, implying lesser efficient use of light (Fig. 3). The seasonal trend of weekly *P*_Cnet_ mimicked the amount of weekly radiation (PAR) received throughout the growing period (Supplementary Fig. S2). It peaked in the weeks around 60 DAE across treatments except for the WS under aCO_2_ peaked about 1–2 weeks earlier. Throughout the season eCO_2_ treatments had higher *P*_Cnet_. Across T regimes, a slightly distinct pattern of relatively enhanced seasonal *P*_Cnet_ in the weeks around 40 DAE was observed for WS treatment under eCO_2_. In fact, a period of slow water depletion (resulting larger water contents in the soil) was also observed for the same treatment (Fig. 1). The eCO_2_ mediated decreases in transpiration while the increase of photosynthesis results in greater water use efficiency that was consistent with the observations made in this study. Conversely, high T increases evapotranspiration and often results in a lower water use efficiency^27^.

### The seasonal canopy photosynthesis and dry mass production was closely related

**Figure 4:**
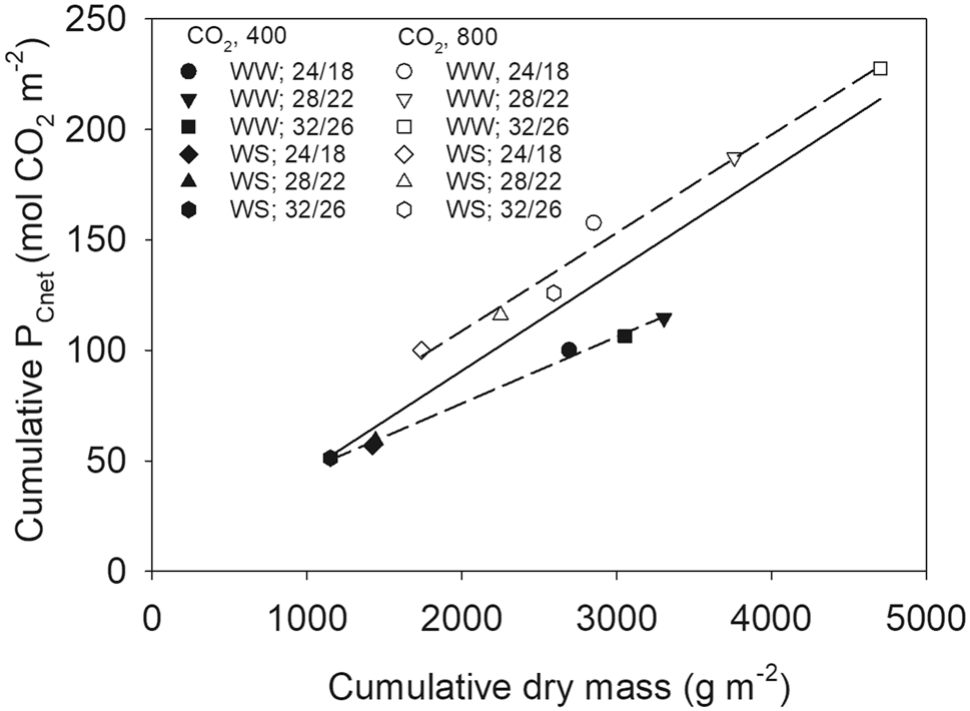
Seasonal cumulative net assimilation versus dry mass production for soybean grown across 12 treatments as two levels of each CO_2_ (400 and 800 µmol mol^−1^) and irrigation (WW, well water; WS, water stress) across three temperature (T, day/night, °C) regimes. Solid line shows a regression fit across all treatments (y = 0.045x, r^2^ = 0.97). Dashed lines show regression at ambient (filled symbols, y = 0.036x, r^2^ = 0.99) or elevated (unfilled symbols, y = 0.050x, r^2^ = 0.99) CO_2_ separately. The cumulative dry mass represents all plants inside the chambers at any given period.

The season total dry mass production (g m^−2^) was regressed with the canopy total *P*_Cnet_, (mol CO_2_ m^−2^) to assess the accuracy of the canopy gas exchange measurement over the season (Fig. 4). There was a strong linear correlation between the season total *P*_Cnet_ and biomass, indicating good accuracy of the CER data. Since the mass of carbon in one mole of CO_2_ is 12.0107, when multiplied with the slope (0.045) from the combined regression it indicated a tissue carbon (C) content of the 0.54 g carbon g^−1^ of dry tissue in the biomass based on the *P*_Cnet_^30,31^. This suggest that potentially 54% of the cumulative above-ground biomass production was accounted for by the carbon fixed via canopy photosynthesis. It seemed an overestimation relative to around 40–45% of carbon in soybean tissues. However, the biomass did not account for the roots (not measured) that can be ≈ 20%^32^. Moreover, seed generally has greater carbon concentration (≈ 50%) than the vegetative tissue in soybean^17^. When estimated by accounting for the 20% partitioning to the root biomass, the re-calculated carbon content was 0.45 g carbon g^−1^. When measured separately for each CO_2_ level, the tissue carbon contents were 43% and 60% at aCO_2_ and eCO_2_, respectively (Fig. 4). Increased tissue carbon concentration under eCO_2_ has been found in the soybean seeds^17^. Moreover, increased carbohydrate content due to greater C:N ratio under eCO_2_ has been observed and might be caused by photosynthetic acclimation and greater nitrogen use efficiency^20,31,33^. A greater tissue carbon content and slope under eCO_2_ versus aCO_2_ have been shown previously and were comparable with studies using canopy CER measurements in several crops including soybean^17,27,28,31,34^.

### Plant growth and dry matter production

**Figure 5:**
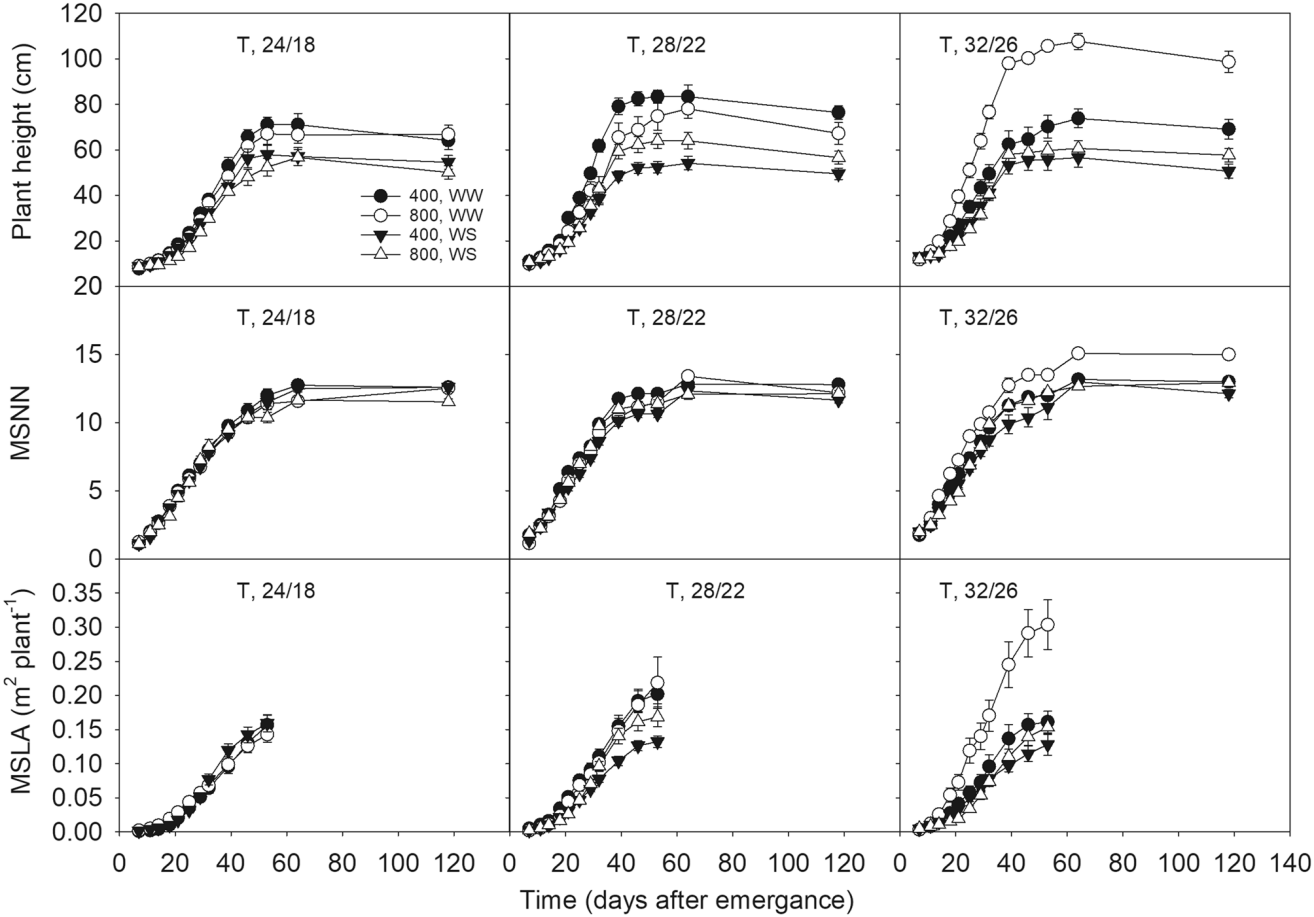
The mainstem plant height (PH), node number, and leaf area of soybean as a function of time across treatments of levels of CO_2_ (400 and 800 µmol mol^−1^) and irrigation (WW, well water; WS, water stress) under three of temperature (T, day/night, °C) regimes. Symbols represent the mean and standard error (when bigger than the symbol) of eight plants.

Plant height and MSNN reached their maximum value around 60 DAE across treatments following sigmoid growth (Fig. 5). A significant I × CO_2_ × T interaction was found for the plant height and node number, SER, and LAER (Table 1). The end season plant height was significantly decreased (15–43%) under WS across all treatments due to a lower rate of stem elongation. Smaller plants under drought often result due to its direct impact on cell division and elongation^18^. Increased plant height and SER at eCO_2_ versus aCO_2_ was also observed under warmer temperatures. The mainstem node number varied between 12 to 15 nodes per plant with the lowest node number observed under WS condition. No significant I × CO_2_ × T interaction was found for NAR (Table 1). A warmer temperature or eCO_2_ increased NAR. However, the negative impact of WS on NAR was primarily observed under aCO_2_. The LAER was negatively impacted by WS whereas eCO_2_ stimulated it with a relatively greater degree under WS than in WW treatment.

**Table 1:** Effects of irrigation (I, WW, well water; WS, water stress), CO_2_ levels (µmol mol^−1^), and temperature (T, day/night, °C) treatments on plant height (PH, cm), mainstem node number (NN), stem elongation rate (SER, cm/day), node elongation rate (NAR, node/day) and leaf area expansion rate (LAER, cm^2^ plant^−1^ day^−1^).

I	CO_2_	T	PH	NN	SER	NAR	LAER
WW	400	22/18	64.1^c,d^	12.6^b,c,d^	1.77^c,d^	0.29^b,c,d^	41.5^d,e,f^
		28/22	76.4^b^	12.8^b,c,d^	2.95^b^	0.34^a,b^	56.9^b,c^
		32/26	69.1^c^	13.0^b^	1.93^c^	0.30^b,c,d^	51.9^b,c,d,e^
	800	22/18	66.8^c^	12.5^b,c,d^	1.63^c,d^	0.27^c,d^	39.6^e,f^
		28/22	67.2^c^	12.2^c,d,e^	1.94^c^	0.33^a,b^	58.8^b^
		32/26	98.7^a^	15.0^a^	3.40^a^	0.31^a,b,c^	87.7^a^
WS	400	22/18	54.5^e,f,g^	12.6^b,c,d^	1.39^d^	0.27^c,d^	36.7^f^
		28/22	49.5^g^	11.7^e^	1.57^c,d^	0.27^c,d^	39.5^e,f^
		32/26	50.7^f,g^	12.1^d,e^	1.61^c,d^	0.26^d^	35.3^f^
	800	22/18	50.1^f,g^	11.5^e^	1.41^d^	0.33^ab^	51.1^b,c,d,e^
		28/22	56.6^e,f^	12.1^d,e^	1.94^c^	0.35^a^	55.2^b,c,d^
		32/26	57.7^d,e^	12.9^b,c^	1.84^c^	0.36^a^	43.7^c,d,e,f^
ANOVA^†^	I		< 0.0001	< 0.0001	< 0.0001	0.8799	< 0.0001
	CO_2_		0.0001	0.1156	0.0470	0.0003	< 0.0001
	T		< 0.0001	< 0.0001	0.0001	0.0746	0.0015
	I × CO_2_		0.1092	0.2439	0.5409	< 0.0001	0.8809
	I × T		< 0.0001	0.0293	0.0052	0.1915	< 0.0001
	CO_2_ × T		< 0.0001	< 0.0001	< 0.0001	0.3577	0.0628
	I × CO_2_ × T		< 0.0001	0.0076	< 0.0001	0.9353	0.0038
**Mean values across each treatment††**							
I treatment	WW		73.7	13.0	2.27	0.31	56.0
	WS		53.2	12.2	1.62	0.31	43.6
CO_2_ treatment	400		60.7	12.5	1.87	0.29*	43.6
	800		66.2	12.7	2.03	0.33	56.0
T treatment	22/18		58.9	12.3	1.55	0.29^B^	42.2
	28/22		62.4	12.2	2.1	0.32^A^	52.6
	32/26		69.0	13.3	2.19	0.31^A,B^	54.7

^†^The significance test (P-values) of the analysis of variance (ANOVA) across treatments and interactions are given. When the I × CO_2_ × T interaction is significant, within a column means followed by the same lowercase letter are not significantly different.

^††^The means across each treatment is also given. When I × CO_2_ × T interaction is not significant, the symbol * indicates a significant difference between values within either I or CO_2_ treatments, and the mean value between T treatments followed by the same uppercase letter is not significant. Data are the mean (n = 8).

Least significant difference at α = 0.05.

Significant I × CO_2_ × T interaction for TLA and TDwt was found at the early (35 DAE) stage of plant growth (Table 2). WS significantly decreased TDwt and TLA regardless of the T or CO_2_ treatments and stage of plants. However, the maximum decrease (46–72%) in TDwt was observed at 119 DAE and at temperatures warmer than OT. The WS induced decreases in TDwt and TLA were also greater under aCO_2_ versus eCO_2_. A positive impact of eCO_2_ on plant biomass accumulation was obvious across T or irrigation regimes. However, the degree of eCO_2_-mediated increase of TDwt was greater at a warmer temperature and under WS treatment relative to the aCO_2_. Moderately warmer temperatures as used in this study have been shown to increase CO_2_ fertilization effects on the biomass accumulation, however, very high temperature or severe heat stress can potentially diminish effect of eCO_2_^12,29,35^.

**Table 2:** Effects of irrigation (I, WW, well water; WS, water stress), CO_2_ levels (µmol mol^−1^), and temperature (T, day/night, °C) treatments on plant total dry weight (TDwt, g plant^−1^), total leaf area (TLA, cm^2^ plant^−1^), number of pods and seeds (plant^−1^), pod and seed dry weight (g plant^−1^), and seed size (g seed^−1^) at 35, 64 and 118 days after emergence (DAE).

Treatment			35 DAE		64 DAE				119 DAE					
I	CO_2_	T	TDwt	TLA	TDwt	TLA	Pods	Pod wt	TDwt	Pods	Pod wt	Seeds	Seed wt	g seed^−1^
WW	400	22/18	6.6^c,d,e^	1334^b,c,d,e^	27.7^b,c,d^	3042^b,c^	63.2^b,c^	6.3^c,d^	82.4^b^	83.1^c,d^	52.2^b^	183.8^b,c^	38.9^a,b^	0.20^b,c^
		28/22	8.8^b^	1689^b^	33.6^bc^	3081^b,c^	71.3^a,b^	9.2^b^	99.9^b^	100.0^b,c^	58.9^b^	217.9^b^	43.2^a^	0.19^c,d^
		32/26	7.6^b,c,d^	1394^b,c,d^	37.0^a,b^	4832^a^	54.2^c^	3.4^e,f^	82.7^b^	128.1^b^	48.2^b,c^	212.2^b^	30.2^b,c^	0.14^h^
	800	22/18	6.4^c,d,e^	1241^c,d,e^	25.9^b,c,d,e^	2295^c,d^	52.2^c,d^	6.2^c,d^	94.8^b^	84.1^c,d^	57.5^b^	188.9^b,c^	42.1^a^	0.22^a^
		28/22	9.1^b^	1522^b,c,d^	47.0^a^	3767^b^	87.7^a^	12.2^a^	100.0^b^	80.6^c,d^	58.5^b^	186.3^b,c^	39.5^a,b^	0.21^b^
		32/26	13.6^a^	2493^a^	48.0^a^	5307^a^	57.9^b,c^	3.9^d,e^	139.2^a^	182.4^a^	77.5^a^	308.1^a^	49.5^a^	0.16^f,g^
WS	400	22/18	4.6^e^	937^e^	16.4^e^	1794^d^	32.5^e^	3.4^e,f^	37.5^d,e^	31.9^f^	18.4^d,e^	75.0^e^	13.7^d,e^	0.19^d^
		28/22	5.8^d,e^	1163^d,e^	17.8^d,e^	1799^d^	36.7^d,e^	5.0^d,e^	32.9^d,e^	39.3^e,f^	19.4^d,e^	83.4^d,e^	13.7^d,e^	0.16^e,f,g^
		32/26	5.1^e^	921^e^	15.1^e^	1961^d^	32.3^e^	1.2^f^	23.4^e^	47.6^e,f^	10.6^e^	69.1^e^	6.6^e^	0.09^i^
	800	22/18	5.1^e^	1109^d,e^	24.8^c,d,e^	2269^c,d^	51.0^c,d^	7.7^b,c^	38.3^d,e^	45.9^e,f^	23.6^d,e^	108.5^d,e^	16.7^d,e^	0.16^ g^
		28/22	8.0^b,c^	1622^b,c^	27.8^b,c,d^	2347^c,d^	51.8^c,d^	7.9^b,c^	53.8^c,d^	58.4^d,e,f^	30.3^d^	114.5^d,e^	20.1^c,d^	0.17^e^
		32/26	6.5^c,d,e^	1220^c,d,e^	28.8^b,c,d^	2634^c,d^	49^c,d,e^	4.1^d,e^	74.9^b,c^	65.2^d,e^	34.3^c,d^	140^c,d^	23.7^c,d^	0.17^ef^
ANOVA^†^	I		< 0.0001	< 0.0001	< 0.0001	< 0.0001	< 0.0001	0.0003	< 0.0001	< 0.0001	< 0.0001	< 0.0001	< 0.0001	< 0.0001
	CO_2_		< 0.0001	0.0013	0.0001	0.0478	0.0049	< 0.0001	< 0.0001	0.0196	0.0002	0.0061	0.0010	< 0.0001
	T		< 0.0001	0.0017	0.0048	< 0.0001	0.0027	< 0.0001	0.0618	< 0.0001	0.4591	0.0133	0.8214	< 0.0001
	I × CO_2_		0.4190	0.8653	0.4866	0.2272	0.0488	0.0369	0.9052	0.6898	0.7750	0.3711	0.5674	0.6273
	I × T		0.0037	0.0046	0.0324	< 0.0001	0.0308	0.0114	0.7345	0.0002	0.6407	0.0929	0.9862	0.001
	CO_2_ × T		0.0026	0.0067	0.2105	0.1503	0.3681	0.6312	0.0013	0.0424	0.0109	0.0162	0.0047	< 0.0001
	I × CO_2_ × T		0.0037	0.0042	0.4871	0.263	0.1972	0.2192	0.4774	0.0413	0.5589	0.3425	0.4948	< 0.0001
**Mean values across each treatment**^††^														
I treatment	WW		8.7	1612	36.5*	3721*	64.4*	6.9*	99.8*	109.7	58.7*	216.2*	40.5*	0.19
	WS		5.9	1162	21.8	2134	42.2	4.9	43.5	48.1	22.8	98.4	15.7	0.16
CO_2_ treatment	400		6.4	1240	24.6*	2751*	48.4*	4.7*	59.8*	71.7	34.6*	140.2*	24.4*	0.16
	800		8.1	1534	33.7	3103	58.3	7.0	83.5	86.1	46.9	174.4	31.9	0.18
T treatment	24/18		5.7	1155	23.7^B^	2350^B^	49.7^B^	5.9^B^	63.2^B^	61.3	37.9	139.1^B^	27.8	0.19
	28/22		7.9	1499	31.6^A^	2748^B^	61.9^A^	8.6^A^	71.7^AB^	69.6	41.8^A^	150.5^B^	29.1	0.18
	32/26		8.2	1507	32.2^A^	3684^A^	48.4^B^	3.1^C^	80.1^A^	105.8	42.6^A^	182.4^A^	27.5	0.14

^†^The significance test (P-values) of the analysis of variance (ANOVA) across treatments and interactions are given. When the I × CO_2_ × T interaction is significant, within a column means followed by the same lowercase letter are not significantly different.

^††^The means across each treatment is also given. When I × CO_2_ × T interaction is not significant, the symbol * indicates a significant difference between values within either I or CO_2_ treatments, and the mean value between T treatments followed by the same uppercase letter is not significant. Data are the mean (n = 18, 12, and 15 for 35, 64 and 119 DAE, respectively).

Least significant difference at α = 0.05.

The number of pods and pod weight per plant increased between 64 and 119 DAE (Table 2). At 119 DAE, the pod and seed weight per plant showed a similar response pattern. WS significantly decreased pod or seed weight on average ≈ 54% and 61% at 64 and 119 DAE, respectively. However, averaged decreased pod or seed weight in WS treatment was lesser under eCO_2_ (54%) versus eCO_2_ (70%) at 119 DAE. A temperature cooler or warmer than the OT resulted in lower pod and seed weight across other (I and CO_2_) treatments except under eCO_2_ and WW conditions. Relative to aCO_2_, the eCO_2_ consistently increased seed yield per plant, especially at the warmer temperature (54%) and under WS (22–200%). In fact, seed yield response to eCO_2_ under WS increased with temperature. The individual seed size (g seed^−1^) showed significant I × CO_2_ × T interaction. Overall, the seed size significantly decreased under WS and warmer than OT treatment. However, eCO_2_ tended to increase seed size across treatments (Table 2).

As the plant aged the biomass partitioning to leaves and stem decreased while reproductive structures such as pod and seed increased (Table 3). Biomass partitioning across traits (leaves, stems, pods, and seeds) showed I × CO_2_ × T interactions. Averaged over temperature and CO_2_, in WS treatments, biomass partitioning tended to be greater in leaves than in the stems. Relative to the WW, the partitioning to leaves increased while stem decreased in WS treatment. At 119 DAE, on average 56% of the total biomass was allocated to the pods across treatments. However, in WS treatment under MHT, the partitioning to pods was 45–47% across CO_2_ levels. Relative to the OT, the MHT consistently showed lower biomass partitioning to seed across all treatments. Similarly, WS also decreased (2–25%) biomass partitioning to the seeds across treatments. The pod set, seed numbers per pods, and seed filling durations are the determinants of the soybean yield, and studies have shown that the remobilizations of the photosynthates play crucial roles during the soybean seed growth^22,36,37^. The soybean photosynthetic capacity and biomass allocation are strongly influenced by temperature, water stress, and elevated CO_2_ where water stress, for example, can cause flower abortion less pod set leading so that the available photosynthetic energy is diverted to filling remaining pods^29,36–38^.

**Table 3:** Effects of irrigation (I, WW, well water; WS, water stress), CO_2_ levels (µmol mol^−1^), and temperature (T, day/night, °C) treatments on the dry mass partition in leaves, stems, pods and seeds (%) at 35, 64 and 118 days after emergence (DAE).

Treatment			35 DAE		64 DAE			119 DAE			
I	CO_2_	T	Leaf	Stem	Leaf	Stem	Pod	Leaf	Stem	Pod	Seed
WW	400	22/18	57.4^b,c^	42.6^f,g^	39.8^d,e^	37.9^c^	22.2^b^	20.6^d^	15.2^d,e,f^	64.2^a^	48.6^a^
		28/22	52.4^g^	47.6^b^	34.7^g,h^	38.5^c^	26.8^a^	23.7^b,c,d^	16.7^c,d^	59.6^b,c,d^	43.9^a,b^
		32/26	54.7^e,f^	45.3^c,d^	44.7^b^	47.1^b^	8.2^d^	23.5^b,c,d^	19.1^b^	57.4^c,d,e^	35.4^d^
	800	22/18	54.9^e^	45.1^d^	39.2^d,e^	38.3^c^	22.5^b^	21.9^c,d^	16.7^b,c^	61.3^a,b,c^	45.2^a,b^
		28/22	52.6^g^	47.4^b^	35.6^f,g,h^	37.7^c^	26.6^a^	25.0^b,c^	16.3^c,d,e^	58.7^b,c,d^	41.0^b,c^
		32/26	45.6^h^	54.4^a^	38.0^e,f^	54.2^a^	7.8^d^	24.3^b,c^	21.7^a^	54.1^e^	34.5^d^
WS	400	22/18	58.4^a,b^	41.6^g,h^	43.9^b,c^	36.6^c^	19.5^b^	37.6^a^	13.5^f^	49.0^f^	36.7^c,d^
		28/22	55.5^d,e^	44.5^d,e^	41.4^c,d^	30.4^d^	28.1^a^	26.5^b^	14.5^e,f^	58.9^b,c,d^	42.0^b^
		32/26	56.5^c,d^	43.5^e,f^	54.0^a^	38.5^c^	7.5^d^	35.9^a^	19.3^b^	44.8^f^	28.2^e^
	800	22/18	59.5^a^	40.5^h^	37.1^e,f,g^	33.2^d^	29.7^a^	23.6^b,c,d^	14.4^e,f^	62.0^a,b^	44.2^a,b^
		28/22	52.4^g^	47.6^b^	32.9^h^	39.3^c^	27.8^a^	26.3^b^	18.1^b,c^	55.6^d,e^	36.0^d^
		32/26	53.3^f,g^	46.7^b,c^	39.6^d,e^	46.4^b^	14.0^c^	36.1^a^	16.9^c,d^	47.0^f^	32.9^d,e^
ANOVA^†^	I		< 0.0001	< 0.0001	< 0.0001	< 0.0001	0.0063	< 0.0001	0.0003	< 0.0001	< 0.0001
	CO_2_		< 0.0001	< 0.0001	< 0.0001	< 0.0001	0.0005	0.0116	0.0156	0.4069	0.8832
	T		< 0.0001	< 0.0001	< 0.0001	< 0.0001	< 0.0001	< 0.0001	< 0.0001	< 0.0001	< 0.0001
	I × CO_2_		0.0010	0.0010	< 0.0001	0.0790	0.0003	< 0.0001	0.5458	0.0011	0.0235
	I × T		0.0002	0.0002	0.006	0.0014	0.7088	< 0.0001	0.0756	0.0026	0.4399
	CO_2_ × T		< 0.0001	< 0.0001	< 0.0001	< 0.0001	0.0121	0.0001	0.2763	0.0056	0.0116
	I × CO_2_ × T		< 0.0001	< 0.0001	0.4969	< 0.0001	0.0205	< 0.0001	< 0.0001	0.0005	0.0116
Mean values across each treatment^††^											
I treatment	WW		52.9	47.1	38.7*	42.3	19.0	23.1	17.7	59.2	41.0
	WS		55.9	44.1	41.5	37.4	21.1	31.0	16.1	52.9	37.1
CO_2_ treatment	400		55.8	44.2	43.1*	38.2	18.7	28.0	16.4	55.6	39.1
	800		53.0	47.0	37.1	41.5	21.4	26.2	17.5	56.4	39.1
T treatment	24/18		57.5	42.5	40.0^B^	36.5	23.5	25.9	14.9	59.1	43.1
	28/22		53.2	46.8	36.1^C^	36.5	27.3	25.4	16.4	58.2	41.5
	32/26		52.5	47.5	44.1^A^	46.5	9.4	30.0	19.2	50.8	32.7

^†^The significance test (P-values) of the analysis of variance (ANOVA) across treatments and interactions are given. When the I × CO_2_ × T interaction is significant, within a column means followed by the same lowercase letter are not significantly different.

^††^The means across each treatment is also given. When I × CO_2_ × T interaction is not significant, the symbol * indicates a significant difference between values within either I or CO_2_ treatments, and the mean value between T treatments followed by the same uppercase letter is not significant. Data are the mean (n = 18, 12, and 15 for 35, 64 and 119 DAE, respectively).

Least significant difference at α = 0.05.

### eCO_2_ enhanced ***P***_Cnet_, dry matter production and seed yield to a greater extent in WS than in the WW treatment

The results showed that, on average across T regimes, within an irrigation treatment, eCO_2_ stimulated about 25% and 90% dry mass under WW and WS, respectively, relative to aCO_2_ (Table 3). The same for seed yield was 21% and 109%, and for *P*_Cnet_ 78% to 105% in WW and WS situations, respectively. In fact, a distinctive linear relationship between cumulative *P*_Cnet_ and dry mass for aCO_2_ and eCO_2_ also indicated a greater conversion of photosynthate into biomass under eCO_2_ (Fig. 4). The current study adds to the general concept that the elevated CO_2_ will enhance soybean productivity. However, recent studies utilizing Free-Air-CO_2_-Enrichment facilities suggested a limited potential of elevated CO_2_ to mitigate the adverse effect of high temperature and/or water stress on soybean growth and yield that strongly depend on the severity of these stresses^4,11,20^. The observed stimulatory effect of eCO_2_ under WS likely have resulted due to less severe drought in the current study. In fact, there was always some water available for the plant to uptake throughout the growing season thus avoiding permanent wilting.

### The impact of WS was more detrimental for ***P***_Cnet_, plant biomass production, and seed yield under the aCO_2_ than in eCO_2_

Averaged across temperature, the WS-mediated decrease the TDwt, *P*_Cnet_, and seed yield ranged between 48 and 70% under aCO_2_ where the same for eCO_2_ was between 40 and 54%. The smaller size (g seed^−1^) of individual seeds also contributed to the decreased yield. Relative to the vegetative processes, the reproductive structures such as flowers and pods and processes including flower fertilization and pollination and seed filling are more sensitive to water stresses^14,36,37^. This finding highlights the beneficial impacts of CO_2_ fertilization under a stress situation of this study. However, in this study, eCO_2_ only partially reduced the adverse impact of WS. Nevertheless, the hypothesis that the impacts of water stress will be more detrimental under ambient than elevated CO_2_ was validated in this experiment. The CO_2_ fertilization can be expected to compensate at least partially under the moderate drought situations when other abiotic factors are non-limiting^11^.

### The warmer than OT showed an increased ***P***_Cnet_, TDwt, and seed yield under eCO_2_ with a greater increase under WW than WS conditions

Relative to the OT, MHT had a positive impact on traits such as *P*_Cnet_, TDwt, and seed yield specifically at eCO_2_ across irrigation regimes. Gary et al.^10,11^ described a beneficial impact of CO_2_ on soybean, however, that is strongly dependent on the severity stress and can occur primarily under moderately warmer temperature or water stress. Moreover, on average, the increase in these traits (*P*_Cnet_, TDwt, and seed yield) under MHT was greater in WW (30%) than in WS (22%) relative to the OT. Thus the second hypothesis, that the moderately warmer temperature will enhance soybean productivity, especially under well-watered conditions was also validated. Ferris et al.^22^ reported about 30% reduction in the soybean seed yield per plant in the combination of eight days of high temperature and drought. However, the high-temperature treatment was + 15 °C greater than the control. Nonetheless, A stimulatory impact of warmer temperature on plant productivity is likely to occur under eCO_2_ provided that factors such as water and nutrient are not severely limiting^6,10^.

## Conclusion

This study highlighted the interactive impacts of three important abiotic factors likely to coexist under natural settings (water, temperature, and CO_2_) on soybean plant productivity. The WS and eCO_2_ both reduced plant water uses by reducing canopy transpiration. However, a substantial increase in water use efficiency was primarily found under eCO_2_. In contrast, the warmer temperature of this study (MHT) consistently increased water use and canopy evapotranspiration while decreasing the water use efficiency across CO_2_ and irrigation treatments. Results showed that canopy photosynthesis, biomass accumulation, and seed yield responded uniquely to the different combinations of treatments. The WS had the greatest impacts on plant productivity across CO_2_ and temperature regimes. However, when WS was applied in combination with eCO_2_, the decreases in traits such as canopy photosynthesis, biomass accumulation, and seed yield were lesser regardless of the temperature treatment. Remarkably, a positive impact of warmer than OT of this study (i.e., MHT) on these traits was also found but only at eCO_2_. This study indicates that CO_2_ fertilization will benefit soybean productivity in a climatic condition with moderately warmer than optimum temperature with limited but consistent water availability.

## Materials and methods

### Experimental facility

Soybean (cv. Ripley, maturity group IV) was planted on June 11, 2018, in the 12 outdoor, sunlit, controlled environment Soil-Plant-Atmosphere-Research (SPAR) chambers at the USDA-ARS facility at Beltsville, MD, USA. Each chamber consists of a steel soilbin (1 m deep by 2 m length by 0.5 m wide; 1.0 m^3^) for plant root growth, which is sealed to a Plexiglas chamber (2.5 m tall by 2.2 m long by 1.4 m wide) to accommodate aerial plant parts and atmospheric conditions, a heating and cooling system, and an environmental monitoring and control system. The Plexiglas transmits > 90% of ambient solar radiation^39^, and chambers are sealed as tightly as possible to minimize gas exchange from outside air. Details of the SPAR chambers and methods of operation and monitoring have been described previously^40,41^. In brief, the air temperature is controlled by cooling and heating of air inside each chamber using chilled ethylene glycol and electrical resistance heaters. The air passes over the cooling coils and heating elements located in the air handling system inside each chamber with a sufficient velocity to cause leaf flutter. The condensate from the cooling coils of each SPAR chamber was automatically collected and weighed every 15 min interval via a pressure transducer and solenoid valves to estimate the canopy evapotranspiration (CET)^42,43^. Each chamber is equipped to measure and control carbon dioxide (CO_2_) inside the SPAR chambers. CO_2_ leakage rates are measured daily by injecting nitrous oxide (N_2_O) and measuring the decay^42^. The continuous monitoring and control of all-important environmental variables including temperature, CO_2_, and irrigation, and plant canopy gas exchange in each chamber are done by a dedicated microcomputer workstation using a custom program^41,42^.

The soilbin of each chamber was filled in layers (≈ 0.15 m thick) with soil containing the mixture of 75% sand and 25% vermiculite (Grace Construction Pro ducts, Cambridge, MA, USA) by volume. Each layer is wet thoroughly as it is added. To measure soil water content, 15 Time Domain Reflectometry (TDR) with 0.30-m-long waveguides (3 rods) were installed horizontally in the soilbin perpendicular to the widest dimension. TDRs were installed at the time of filling soilbin at five depths, replicated at three horizontal positions as described by Ref.^43^.

Each soilbin is equipped with the drip irrigation system using an automated pressure compensated dripper system. Each soilbin had 32 drippers each with a flow rate of 3.78 L/h. connected to four drip emission devices (Xeri-Bird 8 Multi-Outlet, Rain Bird Corporation, AZ, USA) via dripline tubes. All 32 driplines were placed in topsoil using dripper stacks in eight rows ≈ 0.25 m apart along the length of the soilbin. The drippers in each row were spaced ≈ 0.1 m. Dripper system was checked weekly basis randomly in each chamber to ensure proper flow rate. All drippers delivered a total of 2.0 L water/min in each soilbin.

### Treatment and plant culture

Soybean was planted in nine rows (0.2 m apart, five plants/row) in each SPAR chamber. The soil was watered to provide ample moisture for seed germination, and a 28/22 °C day/night temperature (T) was maintained until emergence (7 days after planting). After emergence, 12 treatments combinations consisting two levels of CO_2_, ambient (aCO_2_, 400 µmol mol^−1^) and elevated (eCO_2_, 800 µmol mol^−1^), and two levels of irrigation (I; WW, well water; WS, water stress at 35% of the WW), each under three different temperature (T, day/night, °C) regimes of 24/18 (moderately low, MLT), 28/22 (optimum, OT), and 32/26 (moderately high, MHT) °C were initiated (Supplementary Table S1). The daytime air T was controlled in square-wave fashion and initiated at sunrise and returned to the nighttime temperature 1-h after sunset resulting into 16/8 h. day/night T.

Plants were watered between 9:00 and 10:00 h. once per day through drip-irrigation as a fertigation method using a modified Hoagland’s nutrient solution^44^. At the beginning of experiment 20 g of Osmocote (Osmocote 14-14-14 Fertilizer, Greenhouse Megastore, Danville, IL, USA) was added to the tops soil. The frequency of watering from day to day differed between the irrigation treatments so that the WS treatments would receive about 35% water relative to the WW treatment of the corresponding CO_2_ level for each T regime. The daily irrigation amount was determined based on the daily water use that was calculated from the irrigation amount and the soil water content in each soilbin from the TDR data. The hourly water volumes were averaged by the depth and summed to obtain a total hourly water volume in the soilbins (Fig. 1). Daily water use was calculated as [(previous day TDR-based soil water content + current day total irrigation) − current day TDR-based soil water content]. The TDR based soil water content was obtained between 21:00 and 23:00 h. The late hour TDR data was chosen to minimize water redistribution from the morning hours irrigation and deemed suitable as plants are expected to use the majority of the irrigated water throughout the day. This resulted in an irrigation frequency of once per day for WW chambers and once in three days for WS chamber. The irrigation time on a given day varied as 4–20 min for WW treatments as determined based on the previous day water use and 0–15 min for WS treatments. Controlling irrigation amount resulted in more adequate water supply for the WW treatment without any leakage from each soilbin that was necessary for accurate estimation of the plant water use (Supplementary Table S1 and Fig. 1). In rare incidents when water drained from any of the WW treatment, the water use was not calculated for that day, instead, the value from the previous day was used, depending on the total PAR. Drainage from the WS treatment soils was never observed.

The measured season total irrigation and water use, environmental variables inside each chamber such as T, relative humidity (RH), vapor pressure deficit, and CO_2_ were well within the acceptable range and given in Supplementary Table S1. The vapor pressure deficit (VPD) was estimated according to Murray^45^.

To avoid plant competition and to determine aboveground dry mater components at the early growth periods, two plants from the middle of each row (total 18 plants/chamber) were harvested at 35 days after emergence (DAE), and four rows each with three plants (total 12 plants/chamber) were harvested at 64 DAE per chamber. Thus, five rows (40 cm apart) with three plants each (total 15 plants m^−2^) were retained in each SPAR chamber after 64 DAE leaving 15 plants m^−2^ until the final harvest, which was completed at 119 DAE. The row spacing, plant densities, and harvests were designed to maintain a uniform space around each plant.

### Plant growth measurements

Plant heights, mainstem node numbers, and leaf length at each node were determined on eight marked plants weekly from seven DAE to around 60 DAE. Thereafter the plant height and nod numbers from the destructive harvest were taken and mainstem node number were not measured to avoid plant damage. The leaf length measurements were converted to mainstem leaf areas by developing a relationship between the lengths of different leaves and leaf area measured using a LI-3100 leaf area meter (LICOR, Inc., Lincoln, Nebraska, USA) from the plants during the destructive harvests for each treatment separately. The polynomial second order equation (y = ax^2^ + bx, intercept is set to zero) best described relationships (r^2^ 0.93–0.97) between the individual leaf length and area; where traits a and b are equation coefficients, y is the area in cm^2^, and x is the length in cm. At harvests, each plant was separated into organs (e.g., leaves, stems, pods, seeds) and the leaf area and dry weight were determined after drying at 70 °C in an air-forced oven. All fallen leaves were collected at maturity, dried, and weighed for each chamber and allocated to each plant weighted based on their dry mass. The total dry weight (TDwt) represented the sum of the dry weights of all plant components, except the roots. The soybean flowering stage (R1) were determined by tracking 10 plants according to Fehr et al.^46^. When 50% plants had at least one open flower at any node on the main stem, R1 stage was reported.

### Canopy gas exchange measurements

The CO_2_ exchange rate (CER) during the daytime represents canopy net photosynthesis for the entire plant population in the chamber (*P*_Cnet_, µmol CO_2_ m^−2^ s^−1^) and was derived based on the amount of CO_2_ injected into the chamber and the CO_2_ not used by plants or lost due to CO_2_ leakage^40^. The canopy gross photosynthesis (*P*_gross_) was estimated as *R*_D_ + *P*_Cnet_ where *R*_D_ (µmol CO_2_ m^−2^ s^−1^) is the total respiration. *R*_D_ was calculated from CER values during daytime and night-time temperatures. Daytime calculations were obtained between 21:00 h and 22:00 h each day when solar radiation was negligible, and estimates obtained between 1:00 to 5:00 h were used during night-time temperature as described by Fleisher et al.^31^. The canopy CER data were averaged at 15 min intervals and modeled using the non-linear rectangular hyperbola function^47^:

1 $$Gross\, \, photosynthesis = \frac{{a \times {\text{PAR}} \times P_{{{\text{max}}}} }}{{\left( {a \times {\text{PAR}}} \right) + P_{{{\text{max}}}} }},$$

where *P*_max_ is the asymptotic rate of the gross photosynthesis in µmole CO_2_ m^−2^ s^-1^ at light saturation, PAR is the photosynthetically active radiation (µmol m^−2^ s^−1^), and *a* is the coefficient with the unit of µmol PAR m^−2^ s^−1^. The PROC NLIN procedure of the SAS (SAS Enterprise Guide, 4.2, SAS Institute Inc., NC, USA) was used to obtain these traits by Gauss–Newton iteration method using Eq. (1). This procedure was necessary to smooth the CER data and interpolate missing data when SPAR chamber doors were open to take measurements^40^.

### Data analysis

The relationships of plant height, mainstem nodes, and leaf area with time (DAE) were used to estimate the rates of mainstem elongation (SER), node addition (NAR) and leaf area expansion (LAER), respectively, as described by Singh et al.^48^. Since the rapid growth of these traits was mainly associated with the linear part of the curves residing between 18 and 39 DAE (Fig. 1), the average rate in this period was taken as the representative of the maximum SER, NAR, and LAER.

Statistical analyses were performed using SAS (SAS Enterprise Guide, 4.2, SAS Institute Inc., NC, USA). A prior uniformity study using the same chambers^41^ indicated that no statistical differences for plant growth and photosynthesis traits were present among the SPAR chambers. The Fleisher et al.^41^, paper also outlined the methods for data collections and no significant differences among final chamber means for any plant growth response were found, and most values were within 95% confidence limits of the pooled chamber mean. It was concluded that although some differences in among‐chamber and within‐chamber variability were present, when appropriate experimental protocols are followed and within‐chamber positional effects are taken into account during sampling, effects of inherent differences in chamber performance on plant responses can be reduced. Hence, to test for the effect of treatments and their interaction on growth measurements, PROC MIXED with Kenward–Rogers (kr) adjustment of degrees of freedom was used for the analysis of variance (ANOVA) using individual plants within chambers as pseudoreplicates. Treatments (Irrigation, I; CO_2_; T) and their interactions were considered as fixed effects and individual plants as a random effect. The treatment comparisons were conducted by least square means (LSMEANS) procedure (at α = 0.05) with the letter grouping obtained using pdmix800 macro (Saxton, 1998). The means across each treatment is also given to highlight the differences, especially when treatment interaction (I × CO_2_ × T) was not significant. The regression analysis was conducted using the PROC REG procedure of SAS or SigmaPlot (Version 13.0 Systat Software Inc., CA, USA).

### Ethical statement

All the experiments on plants were carried out in accordance with guidelines of US Department of Agriculture—Agricultural Research Service.

## Supplementary Information

Supplementary Information.

## Abbreviations

DAE: Days after emergence
CET: Canopy evapotranspiration
OT: Optimum temperature
MHT: Moderately high temperature
MLT: Moderately low temperature
LAER: Leaf area expansion rate
NAR: Node addition rate
SER: Main stem elongation rate
MSLA: Main stem leaf area
MSNN: Main stem node number
PAR: Photosynthetically active radiation
*P*_*C*__net_: Canopy net canopy photosynthesis
