# Supplementary material for: Impact of water stress under ambient and elevated carbon dioxide across three temperature regimes on soybean canopy gas exchange and productivity
Source: Sci Rep. 2021 Aug 13;11:16511. doi: 10.1038/s41598-021-96037-9 (PMC8363729; doi:10.1038/s41598-021-96037-9)
Supplement: Supplementary file 1 — Supplementary Information. [file 41598_2021_96037_MOESM1_ESM.pdf]

Supplementary Information

Impact of water stress under ambient and elevated carbon dioxide across three temperature regimes on soybean canopy gas exchange and productivity

Shardendu K. Singh[1,2*], Vangimalla R. Reddy[1], Mura Jyostna Devi[1,3*], Dennis J. Timlin[1]

[1]Adaptive Cropping Systems Laboratory, USDA-ARS, Beltsville, MD, USA

[2]Current address: AeroFarms, Newark, NJ, USA

[3]Vegetable Crops Research Unit, USDA-ARS, Department of Horticulture, University of Wisconsin-Madison, Madison, WI, USA

*Corresponding author: singh.shardendu@gmail.com; Jyostna.mura@usda.gov

Figure S1: The hourly dynamics profile of water volume (L m^{-3}) of soilbin for each of the 12 treatments between 6/26/2018 (eight days after emergence) and the maturity of soybean. The treatment are two levels of CO_2 (400 and 800 µmol mol^{-1}) and irrigation (WW, well water; WS, water stress) across three temperature (T, day/night, °C) regimes.

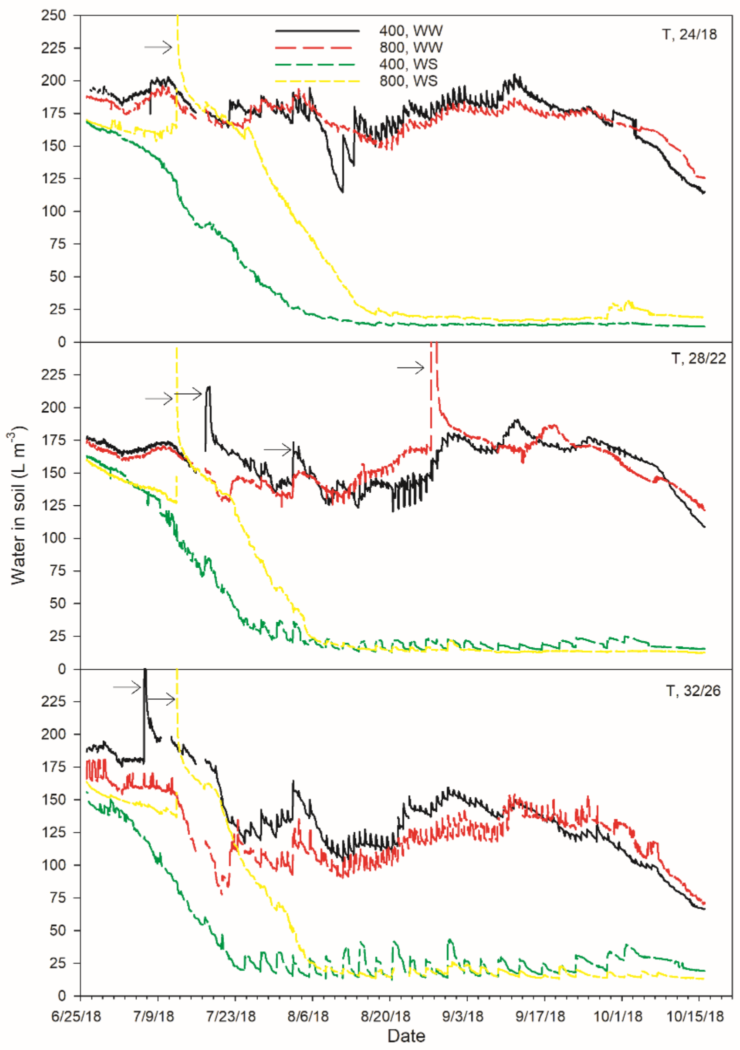

Figure S2: The weekly sum of soybean canopy net photosynthesis (P_{Cnet}) throughout the season as influenced by the treatments of CO_2 levels (400 and 800 μmol mol^{-1}) and irrigation (WW, well water; WS, water stress) under three temperature (T, day/night, °C) regimes. The photosynthetically active radiation (PAR, square symbols) throughout the growing season is also shown (tope panel).

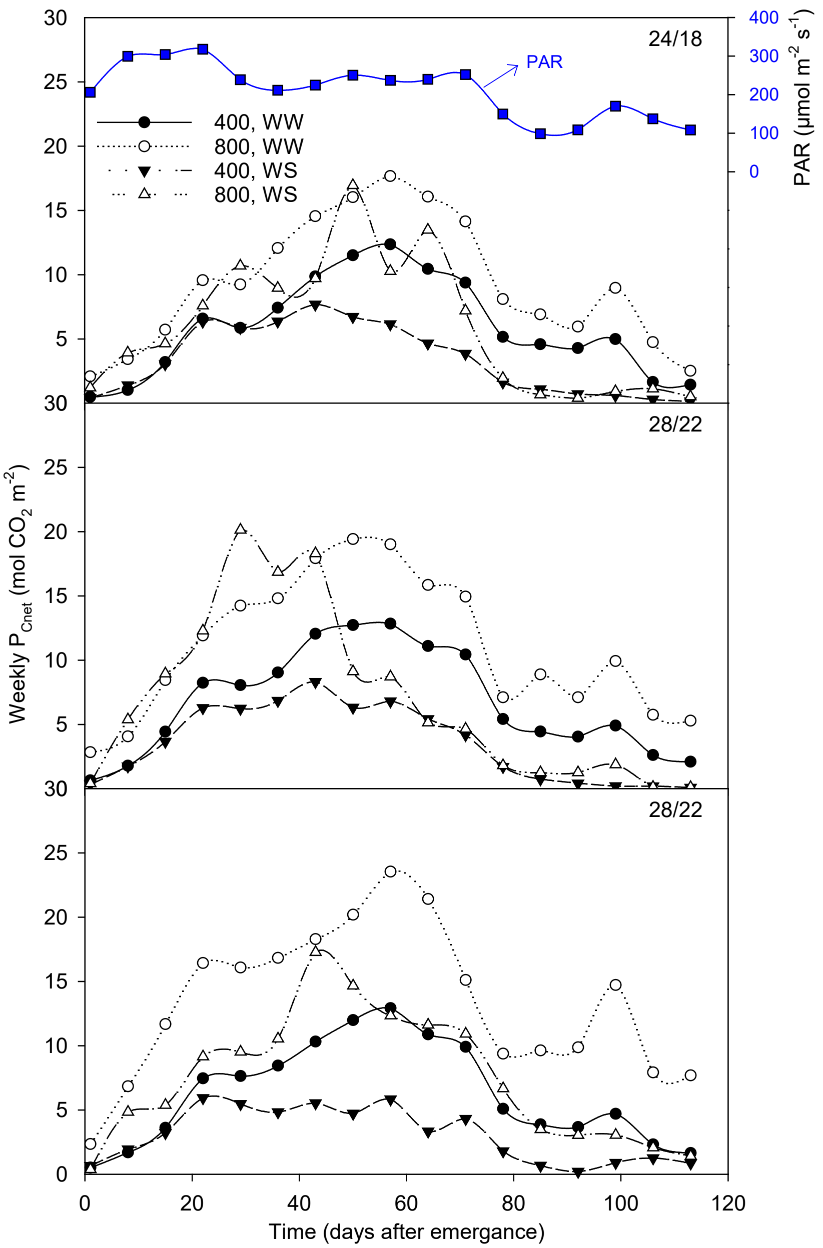

Table S1. The treatment set conditions as irrigation levels (WW, well water, WS, water-stress), CO_2 concentration, and day/night air temperature (T), daily mean T, and the measured values for total seasonal irrigation and water uptake, and seasonal daily mean T, relative humidity (RH), vapor pressure deficit (VPD), and CO_2 concentration inside the chambers. The standard deviation (n = 110) between emergence and maturity is indicated wherever appropriate.

Treatments				Measured seasonal variables					
Irrigation	CO_2 (µmol m^{-2} s^{-1})	T day/night (°C)	Daily Mean T (°C)	Total Irrigation (L)[a,b]	Total uptake (L)	Daily Mean T (°C)	Mean RH (%)[c]	Mean VPD (kPa)[c]	Mean CO_2 (µmol m^{-2} s^{-1})[c]
WW	400	24/18	22	830	895	22.2 ± 0.05	59.6 ± 1.9	1.1 ± 0.05	425 ± 17
		28/22	26	1007	1060	26.0 ± 0.03	53.4 ± 2.4	1.6 ± 0.10	427 ± 17
		32/26	30	1208	1274	30.0 ± 0.09	53.4 ± 3.0	2.0 ± 0.13	427 ± 16
	800	24/18	22	705	722	22.0 ± 0.03	61.7 ± 1.8	1.0 ± 0.05	790 ± 18
		28/22	26	951	860	26.0 ± 0.09	53.0 ± 2.8	1.6 ± 0.12	787 ± 20
		32/26	30	1109	1186	30.0 ± 0.14	55.4 ± 2.3	1.9 ± 0.09	783 ± 19
WS	400	24/18	22	264 (32)	399	22.2 ± 0.14	68.2 ± 2.4	0.9 ± 0.06	412 ± 11
		28/22	26	356 (35)	486	26.1 ± 0.07	56.0 ± 2.9	1.5 ± 0.11	414 ± 13
		32/26	30	424 (35)	538	30.1 ± 0.11	49.3 ± 3.3	2.2 ± 0.15	426 ± 21
	800	24/18	22	252 (36)	417	22.1 ± 0.09	69.2 ± 2.4	0.8 ± 0.07	787 ± 17
		28/22	26	302 (32)	462	26.1 ± 0.06	59.3 ± 3.3	1.4 ± 0.11	785 ± 19
		32/26	30	393 (35)	557	30.0 ± 0.06	49.8 ± 3.0	2.1 ± 0.13	787 ± 16

[a] The value in parenthesis is the total irrigation amount expressed as the percentage of the WW for the corresponding CO_2 treatment at each T regime.

[b] There was already available soil water determined from the TDR system in each soilbin averaging 180 ± 11 and 160 ± 8 L across WW and WS treatments, respectively, which is not included in this column.

[c] daytime values are reported.
